# 

**Published:** 1899-01

**Authors:** 


The Texas State Medical Association will meet this year at San Antonio. The date is the fourth Tuesday in April. The beauty and picturesqueness of the historic old city is only equaled by the warm-hearted hospitality of its citizens and the beauty and grace of its women. Let every Texas doctor make it his especial business to attend this meeting;—he will combine a discharge of duty with the delights of an outing, and will have a royal good time. Let every lover of his profession urge all his brother doctors to attend. Let the 1899 meeting be an epoch-marking one—the reunion of the profession of Texas. Let every endeavor be bent to building up our State organization, nor cease till every reputable physician in Texas is enrolled as a member. Urge at once the organization of the doctors at home. We must begin on the ground and organize all the way up, till it culminates in a grand State association which shall be an honor to the profession and to Texas, and become, as it should be, a power for good. In the matter of better legislation in the interest of the public health every man is expected to do his duty.

